# Interments in the City and Liberties of Philadelphia, from the 11th to the 18th of September

**Published:** 1841-09-25

**Authors:** 


### HEALTH OF THE CITY

Interments *in the City and Liberties of Philadelphia, from the 11th to the 18th of September.*

| Diseases. | Adults. | Children. | Diseases. | Adults. | Children. |
|---|---|---|---|---|---|
| Bowel Complaint, | 0 | 11 | *Brought forward,* | 29 | 51 |
| Cancer, | 1 | 0 | Neglect, | 1 | 0 |
| —— Mouth, | 0 | 1 | Old age, | 4 | 0 |
| Casualty, | 0 | 1 | Palsy, | 1 | 0 |
| Cholera Morbus, | 0 | 1 | Pneumothorax, | 1 | 0 |
| Consumption of | | | Rupture of Uterus, | 1 | 0 |
| the lungs, | 10 | 1 | Small pox, | 0 | 9 |
| Convulsions, | 0 | 6 | Still-born, | 0 | 7 |
| Diarrhœa, | 0 | 3 | Teething, | 0 | 1 |
| Dropsy, | 1 | 0 | Tabes Mesente- | | |
| —— abdominal, | 1 | 0 | rica, | 0 | 1 |
| —— of the | | | Tetanus, | 0 | 1 |
| head, | 0 | 4 | Ulcerated Sore | | |
| —— Breast, | 3 | 0 | Throat, | 0 | 1 |
| Disease of the | | | Unknown, | 1 | 1 |
| Heart, | 1 | 0 | Varioloid, | 0 | 1 |
| —— Bowels, | 0 | 1 | | — | — |
| Drowned, | 1 | 0 | Total, 111— | 38 | 73 |
| Dysentery, | 0 | 1 | —— | | |
| Debility | 1 | 2 | Of the above, there | | |
| Fever, Remittent, | 1 | 0 | were under 1 year, | | 37 |
| —— Nervous, | 1 | 0 | From 1 to 2 | | 19 |
| —— Typhoid, | 0 | 1 | 2 to 5 | | 11 |
| —— Scarlet, | 0 | 2 | 5 to 10 | | 1 |
| Hernia, | 1 | 0 | 10 to 15 | | 4 |
| Inflammation of | | | 15 to 20 | | 1 |
| the Brain, | 1 | 3 | 20 to 30 | | 13 |
| —— Bronchi, | 1 | 0 | 30 to 40 | | 9 |
| —— Lungs, | 1 | 2 | 40 to 50 | | 4 |
| —— Bowels, | 0 | 2 | 50 to 60 | | 3 |
| Marasmus, | 2 | 7 | 60 to 70 | | 2 |
| Malformation, | 0 | 1 | 70 to 80 | | 2 |
| Measles, | 0 | 1 | 80 to 90 | | 3 |
| Mania a Potu, | 2 | 0 | 90 to 100 | | 2 |
| | — | — | | | —— |
| *Carried forward,* | 29 | 51 | Total, | | 111 |

Of the above there were 6 from the almshouse, and 6 people of colour, which are included in the total amount.

